# The R93C Variant of *PCSK9* Reduces the Risk of Premature MI in a Chinese Han Population 

**DOI:** 10.3389/fgene.2022.875269

**Published:** 2022-04-11

**Authors:** Lincheng Yang, Tian Pu, Yan Zhang, Hua Yan, Haiyi Yu, Wei Gao

**Affiliations:** ^1^ Department of Cardiology and Institute of Vascular Medicine, Peking University Third Hospital, NHC Key Laboratory of Cardiovascular Molecular Biology and Regulatory Peptides, Key Laboratory of Molecular Cardiovascular Science, Ministry of Education, Beijing Key Laboratory of Cardiovascular Receptors Research, Beijing, China; ^2^ Department of Cardiology and Institute of Cardiovascular Disease, Peking University First Hospital, NHC Key Laboratory of Cardiovascular Molecular Biology and Regulatory Peptides, Key Laboratory of Molecular Cardiovascular Science, Ministry of Education, Beijing, China; ^3^ Department of Cardiology, Wuhan Asia Heart Hospital, Hubei, China

**Keywords:** pcsk9, premature myocardial infarction, dyslipidemia, single-nucleotide polymorphism, genetic variants

## Abstract

**Background:** Dyslipidemia is a common risk factor for premature myocardial infarction (PMI). Our previous work has shown that single-nucleotide polymorphisms (SNPs) of *LDLR*, *APOB,* and *PCSK9* are associated with dyslipidemia, but how these SNPs correlate with risk for PMI is unknown.

**Objective:** This study aims to evaluate the association between SNPs of *LDLR*, *APOB*, *and PCSK9* and risk of PMI in Chinese Han population.

**Methods:** Two cohorts were established. In Cohort 1 (413 in the PMI group and 1,239 in the control group), SNPs of *APOB*, *LDLR*, and *PCSK9* with minor allele frequency (MAF) > 1%, which has been shown to impact the risk of PMI in a Chinese Han population, were thoroughly examined, and gene–environment interactions were analyzed. A model for PMI risk prediction was developed in Cohort 1 and externally validated in Cohort 2 (577 in the PMI group and 270 in the control group).

**Results:** The distribution of the T allele at the *PCSK9* R93C variant (rs151193009, C > T) was lower in the PMI group than that in the control group (PMI vs*.* Control in Cohort 1, 0.8% vs. 2.3%, *P*_*adjust*_ < 0.05; in Cohort 2, 1.0% vs. 2.4%, *P*_*adjust*_ < 0.05). The T allele at *PCSK9* R93C variant (rs151193009, C > T) reduced the risk of PMI by ∼60% regardless of adjusting for confounding factors (in Cohort 1, adjusted *odds ratio* (*OR*) 0.354, 95% *confidence interval* (*CI*) 0.139–0.900, *p* = 0.029; in Cohort 2, adjusted *OR* 0.394, 95% *CI* 0.157–0.987, *p* = 0.047). No gene–environment interactions were observed between the R93C variant and diabetes/hypertension/smoking in PMI occurrence in this Chinese Han population. Our model showed good performance in predicting the risk of PMI in Cohort 1 (AUC 0.839, 95% *CI* 0.815–0.862, *p* < 0.001) and in an external cohort (AUC 0.840, 95% *CI* 0.810–0.871, *p* < 0.001).

**Conclusions:** The *PCSK9* R93C variant was associated with significantly reduced risk of PMI in the Chinese Han population, and the model we developed performed well in predicting PMI risk in this Chinese Han population.

## Highlights

• The distribution of the T allele of the *PCSK9* R93C variant (C > T) is lower in the PMI group.
• The R93C variant of *PCSK9* reduces the risk of PMI.
• A PMI risk prediction model is constructed based on traditional CHD risk factors and *PCSK9* R93C variant.

## Introduction

Acute myocardial infarction (AMI) is a major cause of death in China, and has affected increasingly younger populations in recent years (Ma et al., 2020). Heart failure and psychological disorders that follow premature myocardial infarction (PMI) significantly affect the quality-adjusted life years (QALYs) of patients, although the morbidity of PMI only accounts for 2–6% of all myocardial infarction (MI) cases (Egred et al., 2005; Echols and O’Connor, 2010; McManus et al., 2011).

Dyslipidemia is an important risk factor for PMI. As shown in the YOUNG-MI study, dyslipidemia, smoking, and hypertension are major risk factors for PMI, among which dyslipidemia is the most important (Singh et al., 2018). Familial hypercholesterolemia (FH) is an independent risk factor of PMI and significantly promotes the risk of recurrence of acute coronary syndromes and decreases the onset age of MI (Nanchen et al., 2015). As previously reported, certain mutations of *LDLR*, *APOB,* and *PCSK9* are closely associated with FH (Cohen et al., 2005). In our previous project, Peking University Health Science Center and the University of Michigan Medical School study of Myocardial Infarction (PUUMA-MI), we identified that some single-nucleotide polymorphisms (SNPs) of these three genes were closely related to abnormal metabolism of blood lipids in a Chinese population, and that the minor allele frequency (MAF) of certain loci in this Chinese population was significantly higher than that in a Caucasian population (Tang et al., 2015). However, the relationships between SNPs and the risk of PMI in the Chinese Han population have not been characterized.

In this study, we analyzed the effects of SNPs of *LDLR*, *APOB,* and *PCSK9* on the risk of PMI in a Chinese Han population not currently undergoing lipid-lowering therapy and evaluated the gene–environment interactions between these SNPs and traditional risk factors for CHD. Finally, we combined the selected SNPs and traditional risk factors for CHD to develop a predictive model to evaluate the risk of PMI.

## Materials and Methods

### Study Population

Two cohorts recruited through clinical care were included in our study. Cohort 1 was a subcohort of the Peking University Health Science Center and the University of Michigan Medical School study of Myocardial Infarction (PUUMA-MI) project designed to analyze the effects of SNPs of *APOB*, *LDLR*, and *PCSK9* on the risk of PMI in the Chinese Han population and was used to develop a predictive model of PMI risk. Cohort 2 was an external cohort for validating the effects of the SNPs on the risk of PMI in the Chinese Han population and the predictive performance of the established model. Premature myocardial infarction was defined according to Adult Treatment Panel III (ATP III) as initial onset age ≤55 in males and ≤65 in females.

Cohort 1: A total of 9,823 cases in the PUUMA-MI project (8) were screened for qualified subjects according to the following criteria and were further divided into a PMI group and a control group. Criteria for inclusion included (Ma et al., 2020): Age ≥18, and Age ≤55 for males and ≤65 for females; (Egred et al., 2005); Chinese Han population (McManus et al., 2011); no previous history of taking any types of lipid-lowering therapies (such as statins, cholesterol absorption inhibitors, probucol, cholic acid chelators, fibrates, nicotinic acids, and high purity fish oil) at enrollment (Echols and O’Connor, 2010); no kinship between enrolled subjects. The exclusion criteria were as follows (Ma et al., 2020): abnormal hepatic function (alanine aminotransferase/aspartate aminotransferase ≥ upper limit of reference value) (Egred et al., 2005); abnormal renal function (serum creatinine ≥ upper limit of reference value) (McManus et al., 2011); thyroid diseases (such as hyperthyroidism, hypothyroidism, and thyroiditis) (Echols and O’Connor, 2010); cancer or cachexia (Singh et al., 2018); cerebrovascular diseases (including stroke and TIA) (Nanchen et al., 2015); peripheral vascular diseases; and (Cohen et al., 2005) incomplete clinical information.

Enrolled subjects were further divided into a PMI group and a control group according to the following criteria. For the PMI group, patients with initial AMI type I confirmed by symptoms, signs, electrocardiograms, myocardial injury markers, and coronary angiography (CAG) were included. Myocardial infarction and AMI type I were defined according to the Third Universal Definition of Myocardial Infarction (Thygesen et al., 2012). For the control group, a 1:3 age-matched PMI group met the following criteria: 1) No stenosis ≥50% in any main coronary arteries, including the left main artery, left anterior descending artery, left circumflex artery, and right coronary artery, as confirmed by CAG or coronary CT scan, and exclusion of individuals with MI or myocardial ischemia as determined by clinical symptoms, electrocardiograms, and myocardial injury markers; 2) no medical history of cardiovascular diseases.

Cohort 2: Subjects hospitalized in the Department of Cardiology, Peking University Third Hospital from January 2013 to December 2018 who underwent CAG and met the same criteria as those from the PUUMA-MI study, were consecutively included and divided into the PMI group and the control group according to the criteria described in Cohort 1.

This study was approved by the Ethics Committee of Peking University (Approval No. IRB00001052-11068). Informed consent was obtained from each patient included in the study.

### Data Collection

Samples from Cohort 1 were collected by the Joint Institute of the PUUMA-MI. In total, 1,652 samples from 4 hospitals were collected: 157 from Peking University First Hospital, 535 from Peking University Third Hospital, 747 from Beijing Shijingshan Hospital, and 213 from Asia Heart Disease Hospital of Wuhan. Medical records were obtained as previously described (Tang et al., 2015). Plasma lipid levels were measured after overnight fasting, total cholesterol (TC) and triglycerides (TGs) were measured using an enzymatic method, and high-density lipoprotein cholesterol (HDL-C) and low-density lipoprotein cholesterol (LDL-C) were measured using a liquid selective detergent method (Tang et al., 2015).

Medical records of subjects from Cohort 2 were obtained from Peking University Third Hospital. General information, previous medical history, family history, medications, blood pressure at admission, height, and weight were collected through the electronic medical record system. Body mass index (BMI) was calculated for each patient. Blood samples were collected from the median cubital vein after fasting (at least 8 h) into an EDTA vacuum anticoagulant tube and a procoagulant tube for DNA extraction and biochemical tests, respectively. The blood samples for DNA extraction were centrifuged, numbered, and stored at –80°C for later use. Biochemical markers, including fasting blood glucose, TC, TGs, HDL-C, and LDL-C, were assessed in the clinical laboratory of Peking University Third Hospital.

The definitions of the risk factors of coronary heart disease were identical to previous description (Tang et al., 2015), including age, gender, body mass index, current smoker, hypertension, dyslipidaemia, diabetes history, and family history of premature coronary heart disease. Dyslipidaemia is defined as having one or more of the following criteria (Ma et al., 2020): TG ≥ 1.7 mmol/L (Egred et al., 2005); HDL-C < 1.04 in males and <1.29 mmol/L in females (McManus et al., 2011); LDL ≥3.4 mmol/L; and (Echols and O’Connor, 2010) already on lipid-lowering drugs.

### DNA Extraction and Single-Nucleotide Polymorphism Genotyping

A QIAamp DNA Blood Mini Kit (QIAGEN, Germany) was used to extract DNA from blood samples. A full-wavelength nucleic acid protein analyzer was used to determine DNA quality. Samples that met the concentration and purity requirements were sent to Beijing Genomics Institute (BGI) for analysis. The SNPs were detected using a HumanExome BeadChip (Illumina, United States). Quality of SNPs for Cohort 1 was determined as previously described (Tang et al., 2015). The genotypes of SNPs in Cohort 2 were determined using mass spectrometry. Sample quality control and SNP detection was performed by BGI. For all tested samples in Cohort 2, a 5% random sample was reciprocally tested, and the reproducibility was 100%.

### Statistical Analysis

Matched subjects for the control group in Cohort 1 were selected by propensity score (matching tolerance, 0.18) using SPSS 22.0 software (IBM, United States). Subsequent statistical analyses were performed using SPSS 22.0 software and R software.

Categorical data are presented as percentages (%) or ratios. Pearson’s chi-squared test or Fisher’s exact test was used for comparisons between different groups. Normally distributed continuous data are presented as the mean ± standard deviation. Non-normally distributed data are shown as the median and IQR. Independent *t* tests or Mann–Whitney *U* tests were used for comparisons between two groups. The odds ratio (OR) was determined using logistic regression, and the 95% confidence interval (CI) was calculated. Univariate and multivariate logistic regression models were used to identify risk factors related to PMI. First, risk factors for CHD and SNP loci that were associated with risk of PMI were included in the univariate logistic regression model analysis. Risk factors with *p* < 0.05 in the univariate analysis were then included in a multivariate logistic regression model and analyzed by the enter method. A predictive model of PMI risks was developed based on the weight of each factor. A receiver operating characteristic (ROC) curve was used to analyze the predictive performance of the model for evaluation of PMI risks in different cohorts. The diagnostic cutoff value, sensitivity, and specificity of the model were obtained by the Youden index. Receiver operating characteristic curves were generated using GraphPad Prism 5.0 software. The synergy index (SI) was calculated to determine the gene–environment interactions between the selected SNP loci and traditional CHD risk factors. In this study, the dominant model was used. *p* values were adjusted for false discovery rate in multiple comparison testing. *p* < 0.05 was considered statistically significant.

Using Plink 1.07 software, the goodness of fit chi-squared test was performed to compare whether the SNP genotypes in each cohort conformed to the law of Hardy–Weinberg equilibrium (HWE). *p* < 0.05 represented failure on the HWE test. To guarantee the power of statistical analysis, only variants with MAF >1% that passed the HWE test were analyzed in this study.

## Results

### Clinical Features of Participants in Different Cohorts

**FIGURE 1:**
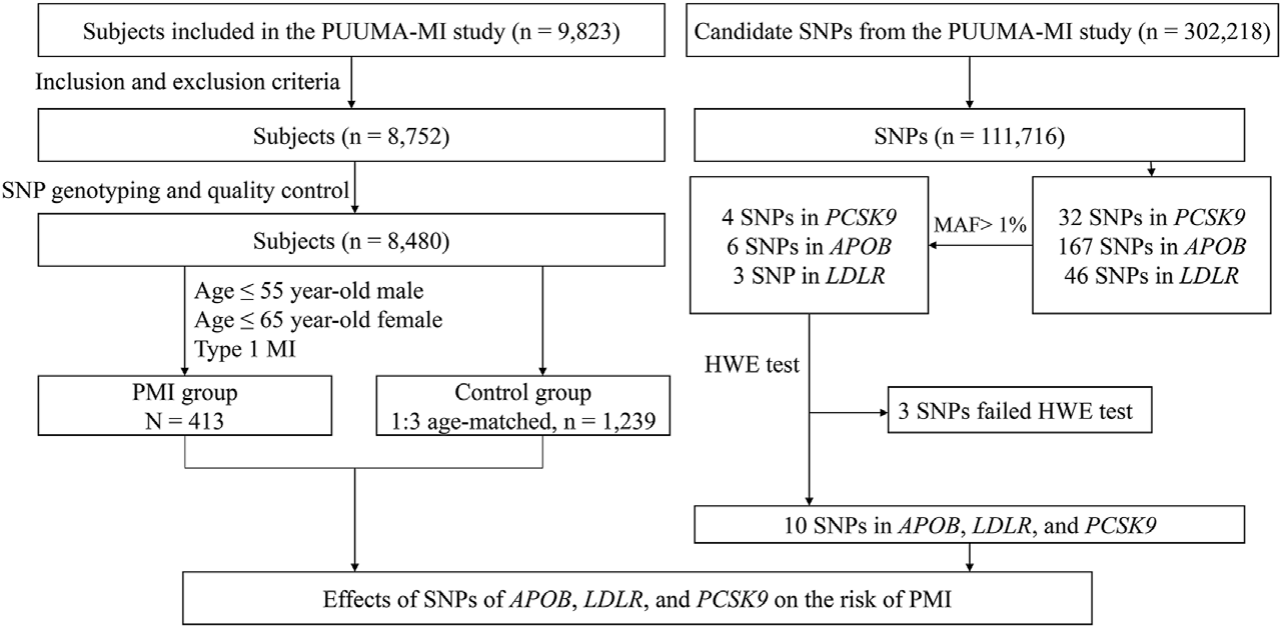
Study flow diagram of Cohort 1. SNP, single-nucleotide polymorphism; PMI, premature myocardial infarction; MAF, minor allele frequency; PCSK9, proprotein convertase subtilisin/kexin type 9; APOB, apolipoprotein B; LDLR, low-density lipoprotein receptor; CHD, coronary heart disease.

A total of 1,652 subjects were enrolled in Cohort 1, including 413 in the PMI group and 1,239 in the control group. A total of 405 (98.1%) subjects in the PMI group had at least one risk factor for CHD, and total of 853 subjects (68.8%) in the control group had at least one risk factor for CHD. The proportions of male patients, and patients with hypertension, diabetes, dyslipidemia, history of smoking, and positive family history of premature CHD were higher in the PMI group than those in the control group (*p* < 0.001). Body Mass Index, TC, TG, and LDL-C were higher in PMI group than those in the control group (*p* < 0.05). In contrast, systolic blood pressure (SBP) and HDL-C were lower in the PMI group than those in the control group (*p* < 0.001). A study flow diagram is presented in Figure 1.

**FIGURE 2:**
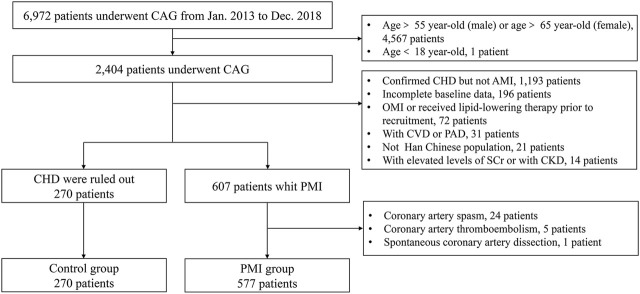
Study flow diagram of Cohort 2. CAG, coronary angiography; AMI, acute myocardial infarction, CHD, coronary heart disease, OMI, old myocardial infarction; CVD, cerebrovascular disease; PAD, peripheral arterial disease; SCr, serum creatinine; CKD, chronic kidney disease.

A total of 847 subjects were enrolled in Cohort 2, including 577 in the PMI group and 270 in the control group. A total of 568 patients (98.4%) in the PMI group had at least one risk factor for CHD, and a total of 206 subjects (76.3%) in the control group had at least one risk factor for CHD. The proportions of male patients, patients with a history of smoking, positive family history of premature CHD were higher in the PMI group than those in the control group. Furthermore, BMI, TC, TG, and LDL-C were higher in the PMI group than those in the control group (*p* < 0.05). The levels of HDL-C in the PMI group were lower than those in the control group (*p* < 0.001). Details are shown in Table 1. A study flow diagram is shown in Figure 2.

**TABLE 1:** Demographics and baseline characteristics of the two cohorts in this study.

	Cohort 1				Cohort 2			
	Control	PMI	*t/Z/Χ* ^*2*^ value	*p* value	Control	PMI	*t/Z/Χ* ^*2*^ value	*p* value
Number, n	1,239	413	–	–	270	577	–	–
Age, years	50.0 ± 6.9	49.8 ± 6.8	0.312	0.755	53.0 (48.8, 59.0)	49.0 (44.0, 54.0)	–7.296	<0.001
Male, n (%)	522 (42.1)	302 (73.1)	119.013	<0.001	116 (43.0)	477 (82.7)	137.738	<0.001
HTN, n (%)	435 (35.1)	240 (58.1)	67.823	<0.001	133 (49.3)	294 (51.0)	0.211	0.646
Diabetes, n (%)	115 (9.3)	107 (25.9)	73.610	<0.001	51 (18.9)	123 (21.3)	0.664	0.415
Dyslipidemia, n (%)	411 (33.2)	312 (75.5)	225.971	<0.001	75 (27.8)	479 (83.0)	3.786	0.052
Smoking, n (%)	377 (30.4)	280 (67.8)	180.577	<0.001	86 (31.9)	407 (70.5)	113.153	<0.001
Family History of PCHD, n (%)	25 (2.0)	62 (15.0)	104.835	<0.001	17 (6.3)	78 (13.5)	9.634	0.002
SBP (mmHg)	133.7 ± 18.0	128.4 ± 17.3	5.202	<0.001	129.0 (119.0, 139.0)	127.0 (115.0, 141.0)	–0.833	0.405
DBP (mmHg)	79.8 ± 10.1	80.2 ± 44.5	–0.305	0.671	80.0 (70.0, 87.0)	80.0 (70.0, 89.0)	–0.522	0.602
BMI (kg/m^2^)	22.6 ± 5.4	26.6 ± 3.2	–11.638	<0.001	24.9 ± 6.7	26.6 ± 8.0	3.056	0.002
TC (mmol/L)	3.90 (3.20, 4.70)	4.50 (3.80, 5.20)	–9.299	<0.001	4.46 ± 1.00	4.61 ± 1.06	–1.921	0.055
TG (mmol/L)	1.42 (1.08, 2.00)	1.60 (1.20, 2.50)	–5.012	<0.001	1.53 (1.03, 2.07)	1.87 (1.41, 2.67)	–5.685	<0.001
HDL-C (mmol/L)	1.31 (1.07, 1.62)	0.90 (0.80, 1.10)	–18.145	<0.001	1.09 (0.93, 1.29)	0.90 (0.79, 1.04)	–3.727	<0.001
LDL-C (mmol/L)	2.60 (2.04, 3.20)	2.80 (2.20, 3.40)	–2.638	0.008	2.67 (2.10, 3.21)	2.88 (2.28, 3.50)	–10.800	<0.001

HTN, hypertension; PCHD, premature coronary heart disease; SBP, systolic blood pressure; DBP, diastolic blood pressure; BMI, body mass index; TC, total cholesterol; TG, triglyceride; HDL-C, high-density lipoprotein cholesterol; LDL-C, low-density lipoprotein cholesterol.

### Distribution of Genotypes and Alleles of SNPs of *PCSK9, APOB,* and *LDLR* in Cohort 1

In Cohort 1, 111,716 SNP loci passed SNP quality control, 243 of which were located in *PCSK9*, *APOB,* and *LDLR*. There were 32 SNPs in *PCSK9,* 4 of which had MAF >1%: rs11583680, rs505151, rs151193009, and rs2479409. There were 167 SNPs in *APOB,* 6 of which had MAF >1%: rs1042034, rs13306198, rs1367117, rs676210, rs679899, and rs13306194. There were 46 SNPs in *LDLR,* 3 of which had MAF >1%: rs6511721, rs2738446, and rs2738459. Therefore, 13 SNPs were subjected to the HWE test, of which 3 failed (*p* < 0.05). These 3 SNPs were excluded from further analysis. Details of the distributions of genotypes of the SNPs of *PCSK9*, *APOB,* or *LDLR* are shown in Supplementary Table S1. The workflow of loci screening is shown in Figure 1.

In Cohort 1, the distribution of the T allele at the *PCSK9* R93C variant (rs151193009, C > T) was lower in the PMI group than that in the control group (PMI vs. Control: 0.8% vs. 2.3%, *P*_*adjust*_ < 0.05), and the distribution of the A allele at the *APOB* R532W variant (rs13306194, G > A) was also lower in the PMI group than that in the control group (PMI vs. Control: 9.3% vs. 12.8%, *P*_*adjust*_ < 0.05). However, the distribution of alleles of other SNPs in the two groups did not differ significantly. Details are shown in Table 2.

**TABLE 2:** Distribution of alleles and the association between different SNPs and incidence of PMI in Cohort 1 in the dominant model.

Gene	SNP	Allele	Control group n (%)	PMI group n (%)	*P*_*adjust*_ for distribution	*OR* (95% *CI*)	*p* value	Adjust *OR* (95% *CI*)^a^	*p* value
*PCSK9*	rs2479409	G	1,731 (69.9)	571 (69.1)	0.932	–	–	–	–
		A	747 (30.1)	255 (30.9)					
	rs151193009	C	2,420 (97.7)	819 (99.2)	0.030	0.402 (0.181–0.892)	0.025	0.354 (0.139–0.900)	0.029
		T	58 (2.3)	7 (0.8)					
	rs505151	G	2,330 (94.0)	778 (94.2)	0.932	–	–	–	–
		A	148 (6.0)	48 (5.8)					
*APOB*	rs1042034	C	1,817 (73.3)	636 (77.0)	0.130	–	–	–	–
		T	661 (26.7)	190 (23.0)					
	rs679899	G	2,075 (83.7)	693 (83.9)	0.932	–	–	–	–
		A	403 (16.3)	133 (16.1)					
	rs13306194	G	2,160 (87.2)	749 (90.7)	0.030	0.664 (0.499–0.883)	0.005	0.813 (0.580–1.140)	0.230
		A	318 (12.8)	77 (9.3)					
	rs13306198	G	2,308 (93.1)	772 (93.5)	0.932	–	–	–	–
		A	170 (6.9)	54 (6.5)					
	rs1367117	G	2,166 (87.4)	712 (86.2)	0.738	–	–	–	–
		A	312 (12.6)	114 (13.8)					
*LDLR*	rs6511721	G	1,848 (74.6)	612 (74.1)	0.932	–	–	–	–
		A	630 (25.4)	214 (25.9)					
	rs2738446	C	2,075 (83.7)	679 (82.2)	0.738	–	–	–	–
		G	403 (16.3)	147 (17.8)					

a Adjusted for age, sex, hypertension, diabetes, dyslipidemia, smoking and premature CHD, family history; SNP, single-nucleotide polymorphism; MAF, minor allele frequency; PMI, premature myocardial infarction, PCSK9 = proprotein convertase subtilisin/kexin type 9, APOB, apolipoprotein B, LDLR, low-density lipoprotein receptor.

### Effects of Single-Nucleotide Polymorphisms of *PCSK9, APOB,* and *LDLR* on the Risk of Premature Myocardial Infarction in the Chinese Han Population

Logistic regression analysis of Cohort 1 showed that the C > T variant (adjusted *OR* 0.354, *95% CI* 0.139–0.900, *p* = 0.029) of *PCSK9* R93C was significantly associated with reduced risk of PMI in Chinese Han individuals not taking lipid-lowering medications. Other SNPs showed no association with the risk of PMI in the Chinese Han population after adjusting for confounding factors. Details are shown in Table 2.

As shown in Table 3, *PCSK9* R93C passed the HWE test (*p* > 0.05) in the PMI and control groups in Cohort 2. We also found that the distribution of the T allele at the PCSK9 R93C was lower in the PMI group than that in the control group in Cohort 2 (PMI vs. Control: 1.0% vs. 2.4%, *P*_*adjust*_ < 0.05), and the C > T variant of *PCSK9* R93C was significantly associated with reduced risk of PMI, with a decrease of ∼60% (*OR* 0.420, 95% *CI* 0.189–0.933, *p* = 0.033; adjusted *OR* 0.394, 95% *CI* 0.157–0.987, *p* = 0.047) in the Chinese Han population, as determined using logistic regression. This result was significant regardless of whether the data were adjusted for confounding factors. These results were consistent with those from Cohort 1. Details are shown in Table 4.

**TABLE 3:** Distribution of genotypes of *PCSK9* rs151193009 and Hardy–Weinberg equilibrium tests in Cohort 2

	Frequency	Numbers of each genotypes, n			*p* value
		CC	CT	TT	
PMI group	Actual frequency	565	12	0	0.800
	Theoretical frequency	565	11	1	
Control group	Actual frequency	257	13	0	0.685
	Theoretical frequency	257	12	1	

PMI, premature myocardial infarction.

**TABLE 4:** Association between rs151193009 and incidence of PMI in Cohort 2 in the dominant model.

SNP	Mutant allele	Mutant allele frequency (%)	Other allele	*OR* (95% *CI*)	*p* value	Adjust *OR* (95% *CI*)^a^	*p* value
rs151193009	T	1.5	C	0.420 (0.189–0.933)	0.033	0.394 (0.157–0.987)	0.047

a Adjusted for age, sex, premature CHD, family history, hypertension, diabetes, dyslipidemia, and smoking.

### Gene–Environment Interactions Between the R93C Variant of *PCSK9* and Traditional Risk Factors for CHD

Gene–environment interactions between the *PCSK9* R93C variant and traditional risk factors for CHD were analyzed. There was a trend toward a synergistic interaction between the variant and diabetes in PMI occurrence (*SI* = 2.769), and an antagonistic interaction between hypertension/smoking and PMI occurrence (with *SI* of 0.413 and 0.120, respectively) in the Chinese Han population. However, none of the *SI*s was statistically significant. Details are shown in Supplementary Table S2.

### Predictive Model for Risk of PMI in the Chinese Han Population Based on Traditional Risk Factors for CHD and the R93C Variant of *PCSK9*

Univariate logistic regression analysis of Cohort 1 showed that six risk factors for CHD apart from age (sex, hypertension, diabetes, hyperlipidemia, smoking, and family history of premature CHD) were closely related to the prevalence of PMI in the Chinese Han population. Multivariate logistic regression analysis of these six variables and *PCSK9* R93C was used to develop a 7-variable predictive model of PMI risks in the Chinese Han population. The weight of each variable in the model is shown in Table 5.

**TABLE 5:** Logistic regression analysis of factors for PMI in Cohort 1

	Univariate analysis		Multivariate analysis (Enter)		
	*OR* (95% *CI*)	*p* value	*OR* (95% *CI*)	*p* value	Weight
Age (1-year-old)	0.998 (0.981–1.014)	0.764	–	–	–
Sex (male vs. female)	3.737 (2.925–4.775)	<0.001	2.221 (1.673–2.948)	<0.001	0.798
HTN (yes vs. no)	2.564 (2.042–3.220)	<0.001	1.950 (1.487–2.558)	<0.001	0.668
Diabetes (yes vs. no)	3.418 (2.552–4.577)	<0.001	2.606 (1.836–3.698)	<0.001	0.958
Dyslipidemia (yes vs. no)	6.223 (4.829–8.020)	<0.001	3.805 (2.871–5.042)	<0.001	1.336
Smoking (yes vs. no)	4.814 (3.789–6.115)	<0.001	3.654 (2.776–4.810)	<0.001	1.296
Family history of PCHD (yes vs. no)	8.578 (5.311–13.852)	<0.001	5.534 (3.143–9.744)	<0.001	1.711
rs151193009 variant (yes vs. no)	0.402 (0.181–0.892)	0.025	0.343 (0.134–0.874)	0.025	–1.070

HTN, hypertension; PCHD, premature coronary heart disease, PCSK9 = proprotein convertase subtilisin/kexin type 9, OR, odds ratio; CI, confidence interval.

**FIGURE 3:**
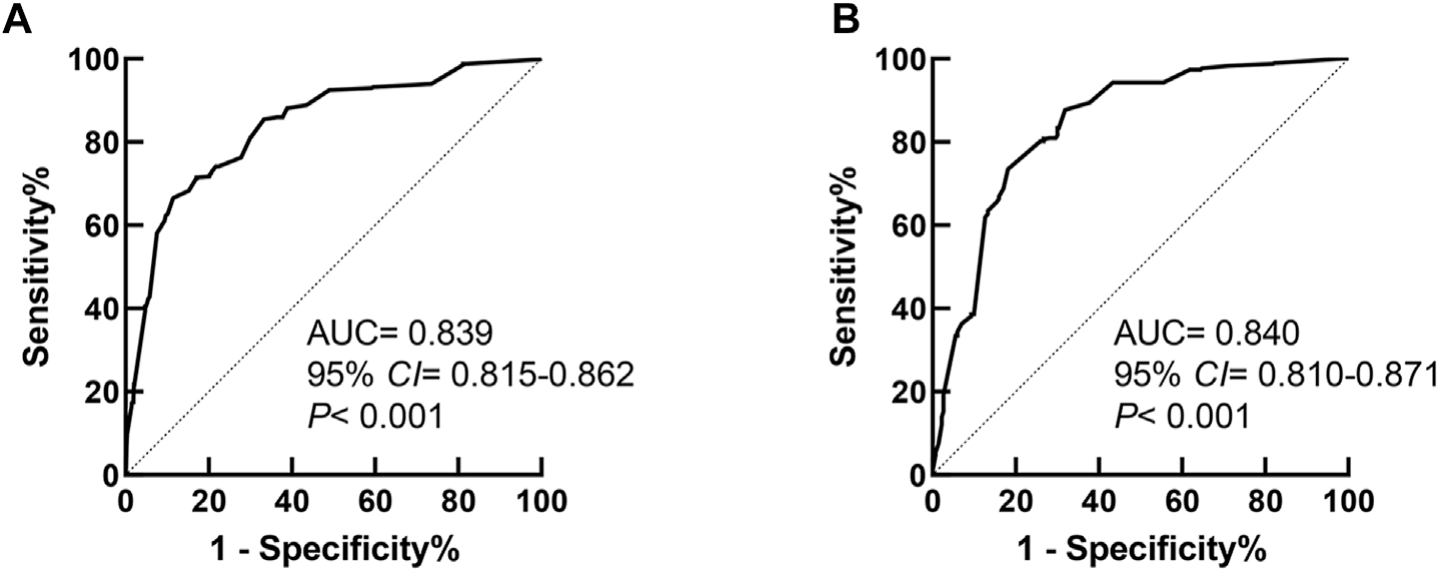
Receiver operator characteristic curve of the prediction model for PMI in Cohort 1 and Cohort 2. **(A)** The ROC curve of the prediction model in Cohort 1. **(B)** The ROC curve of the prediction model in Cohort 2.

Receiver operator curves were generated to test the predictive ability of the model, as illustrated in Figure 3A. The area under the curve (AUC) of the model was 0.839 (95% *CI* 0.815–0.862, *p* < 0.001), which suggested that the model showed good predictive performance for PMI risks in the Chinese Han population. The cutoff value of the model was 2.862, and the Youden index was 0.550 (sensitivity 66.6%, specificity 88.4%).

### External Validation of the Prediction Model in the Chinese Han Population

The performance of the prediction model was validated using Cohort 2 as an external cohort. The ROC curve of the predictive model applied to Cohort 2 is shown in Figure 3B. The AUC of the model was 0.840 (95% *CI* 0.810–0.871, *p* < 0.001), which suggested that the model also had good predictive power for PMI risks in the Chinese Han population in an external cohort.

## Discussion

Based on the results of the PUUMA-MI project, we searched for SNP loci that were associated with risk of PMI in the Chinese Han population through a case-control study in two independent study cohorts. Traditional risk factors for CHD and SNP loci were used to develop a predictive model for PMI risks in the Chinese Han population, the performance of which was validated in an external cohort. The following conclusions were drawn: 1) The R93C variant of *PCSK9* was significantly associated with reduced risk of PMI in the Chinese Han population. 2) The model generated using traditional risk factors for CHD and *PCSK9* R93C had good predictive power for determination of risk of PMI in the Chinese Han population.

The predictive model of PMI risk in the Chinese Han population developed in this study contained 7 variables. These 7 variables included *PCSK9* R93C, and 6 variables (sex, hypertension, diabetes, hyperlipidemia, smoking, family history of premature CHD) that are traditional risk factors for CHD. Information regarding these 6 variables was obtained directly through medical history and physical examination. In the two cohorts of this study, 98.1% and 98.4% of patients with PMI had at least one traditional risk factor for CHD, which was similar to the results of previous studies. (Shah et al., 2016).

Among the six traditional risk factors, family history of premature CHD, dyslipidemia, and smoking contributed most to the model, which suggested that they may play important roles in the pathogenesis of PMI in the Chinese Han population. Previous studies have shown that family history of CHD was an important risk factor for PMI in younger individuals, and approximately 41–71% of patients with PMI have a family history of CHD and premature CHD (Shah et al., 2016). In the two cohorts of this study, the proportions of subjects with a family history of premature CHD in the PMI group were 15.0% and 13.5%, which were lower than those previously reported. However, we found that in the Chinese Han population, a family history of premature CHD increased the risk of PMI approximately 4-fold (*OR* 5.534, 95% *CI* 3.143–9.744, *p* < 0.001), and the OR value was higher than that reported by Oliveira et al., (Oliveira et al., 2009), which suggested that the Chinese Han population may have higher genetic susceptibility to PMI. In this study, more than 70% of patients with PMI suffered from dyslipidemia, which was similar to the findings from previous studies (Chan et al., 2012). A large proportion of patients with PMI and dyslipidemia may have undiagnosed familial hypercholesterolemia. These patients exhibit abnormal metabolism of endogenous cholesterol, and are exposed to hypercholesterolemia from birth, which significantly reduced age of first onset of myocardial infarction. (Wiesbauer et al., 2009; Nanchen et al., 2015). Finally, we also found that smoking was a risk factor for PMI in the Chinese Han population (*OR* 3.654, 95% *CI* 2.776–4.810, *p* < 0.001), and the OR value of smoking in this study was similar to that in previous studies (Yusuf et al., 2004). As previously reported, smoking was one of the most important risk factors associated with PMI (Yusuf et al., 2004; Aggarwal et al., 2012). Although patients with PMI may have a shorter history of smoking, the average daily smoking amount was higher than that of older patients with MI. Continuous and intensive intake of harmful substances may also significantly increase the risk of myocardial infarction (Shah et al., 2016).

The R93C SNP of *PCSK9* is a loss-of-function variant that may reduce the risk of PMI in the Chinese Han population through multiple mechanisms. We found that *PCSK9* R93C was negatively associated with levels of TC and LDL-C (Supplementary Table S3), which indicated that *PCSK9* R93C might be a loss-of-function variant, which was consistent with the findings of previous studies. (Miyake et al., 2008; Tajima et al., 2018; Koyama et al., 2020). Le et al. (Le et al., 2015) found that the Huh7 cell line carrying the *PCSK9* R93C variant generates lower levels of mature PCSK9, which indicated that the variant may affect the generation of mature PCSK9, which may further contribute to loss of function. Although the mechanism underlying how *PCSK9* R93C is involved in reducing PMI risks remains elusive, there are several possible explanations: 1) The variant may directly delay the occurrence and development of atherosclerosis by reducing LDL-C levels; 2) PCSK9 participates in the onset and progression of atherosclerosis by promoting inflammation and macrophage apoptosis. (Tang et al., 2012; Momtazi-Borojeni et al., 2019). The *PCSK9* R93C variant may affect the maturation of PCSK9, weaken its proinflammatory and proapoptotic effects, and indirectly delay the onset and progression of atherosclerosis; 3) Hypertension and smoking are risk factors for PMI (Singh et al., 2018). We found that the *PCSK9* R93C variant might negatively interact with hypertension and smoking, resulting in reduced impact of other CHD risk factors on PMI. In addition, it is a variant specific to Asian populations, which may reduce genetic susceptibility to PMI in Asian populations (Genomes Project et al., 2012; Tang et al., 2015).

Previous studies of lipid metabolism gene variation did not strictly exclude patients who received lipid-lowering treatment prior to recruitment, but only statistically corrected for the effects of lipid-lowering drugs. This approach may not allow for estimation of the true blood lipid levels in the absence of lipid-lowering treatments. In addition, lipid-lowering drugs can regulate *PCSK9* gene expression. Statins can promote the binding of sterol responsive element binding protein 2 (SREBP2) to sterol regulatory elements (SREs) in the promoter region of the *PCSK9* gene, resulting in positive regulation of transcription of the *PCSK9* gene and increased expression of PCSK9 (Lambert et al., 2008). Fibrates can also affect the expression of *PCSK9* by activating peroxisome proliferator activated receptor alpha (PPARα) (Dubuc et al., 2004). These effects may interfere with phenotypic changes caused by *PCSK9* gene mutation. Therefore, use of lipid-lowering drugs may affect the accuracy and reliability of research results in which there are significant effects associated with dyslipidemia. Our study only included patients who did not receive lipid-lowering therapy prior to recruitment, which eliminated the potentially confounding effects of lipid-lowering drugs on the *PCSK9* gene mutation effect.

Our study suffered from several limitations. First, we only analyzed variants with MAF >1% that passed the HWE test to guarantee the power of the statistical analysis, which led to omission of pathogenic variants with MAF <1%. Second, we only analyzed the dominant model due to the limited number of homozygotes. Finally, we only identified the influence of *PCSK9* R93C on PMI risks in the Chinese Han individuals naive to lipid-lowering therapy, so further genetic evidence is needed to confirm that it is an independent risk factor for susceptibility to PMI.

## Data Availability

The datasets presented in this study can be found in online repositories. The names of the repository/repositories and accession number(s) can be found in the article/Supplementary Material.
