# Supplementary material for: The R93C Variant of PCSK9 Reduces the Risk of Premature MI in a Chinese Han Population
Source: Front Genet. 2022 Apr 11;13:875269. doi: 10.3389/fgene.2022.875269 (PMC9035790; doi:10.3389/fgene.2022.875269)
Supplement: Supplementary file 1 [file DataSheet1.docx]

**Table S1. Distribution of genotypes and Hardy–Weinberg equilibrium test of different SNPs in Cohort 1**

| **Gene** | **SNP** | **Genotype** | **Control group, n (%)** | **PMI group, n (%)** | **H–W *P* for**  **control group** | **H–W *P* for PMI group** | ***P* for distribution** | ***P_adjust_*for**  **distribution** |
| --- | --- | --- | --- | --- | --- | --- | --- | --- |
| ***PCSK9*** | rs2479409 | GG | 598 (48.3) | 196 (47.5) | 0.374 | 0.754 | 0.908 | 0.927 |
|  |  | GA | 535 (43.2) | 179 (43.3) |  |  |  |  |
|  |  | AA | 106 (8.6) | 38 (9.2) |  |  |  |  |
|  | rs11583680 | CC | 952 (76.8) | 324 (78.5) | < 0.001 | 0.274 | – | – |
|  |  | CT | 246 (19.9) | 81 (19.6) |  |  |  |  |
|  |  | TT | 41 (3.3) | 8 (1.9) |  |  |  |  |
|  | rs151193009 | CC | 1183 (95.5) | 406 (98.3) | 0.100 | 0.862 | 0.032 | 0.104 |
|  |  | CT | 54 (4.3) | 7 (1.7) |  |  |  |  |
|  |  | TT | 2 (0.2) | 0 (0.0) |  |  |  |  |
|  | rs505151 | GG | 1093 (88.2) | 366 (88.6) | 0.229 | 0.674 | 0.914 | 0.927 |
|  |  | GA | 144 (11.6) | 46 (11.1) |  |  |  |  |
|  |  | AA | 2 (0.2) | 1 (0.2) |  |  |  |  |
| ***APOB*** | rs1042034 | CC | 663 (53.5) | 252 (61.0) | 0.646 | 0.091 | 0.018 | 0.078 |
|  |  | CT | 491 (39.6) | 132 (32.0) |  |  |  |  |
|  |  | TT | 85 (6.9) | 29 (7.0) |  |  |  |  |
|  | rs676210 | GG | 746 (60.2) | 247 (59.8) | < 0.001 | 0.103 | – | – |
|  |  | GA | 493 (39.8) | 137 (33.2) |  |  |  |  |
|  |  | AA | 0 (0.0) | 29 (7.0) |  |  |  |  |
|  | rs679899 | GG | 867 (70.0) | 291 (70.5) | 0.712 | 0.915 | 0.927 | 0.927 |
|  |  | GA | 341 (27.5) | 111 (26.9) |  |  |  |  |
|  |  | AA | 31 (2.4) | 11 (2.7) |  |  |  |  |
|  | rs13306194 | GG | 940(75.9) | 341 (82.6) | 0.721 | 0.707 | 0.018 | 0.078 |
|  |  | GA | 280 (22.6) | 67 (16.2) |  |  |  |  |
|  |  | AA | 19 (1.5) | 5 (1.2) |  |  |  |  |
|  | rs13306198 | GG | 1076 (86.8) | 360 (87.2) | 0.603 | 0.538 | 0.715 | 0.927 |
|  |  | GA | 156 (12.6) | 52 (12.6) |  |  |  |  |
|  |  | AA | 7 (0.6) | 1 (0.2) |  |  |  |  |
|  | rs1367117 | GG | 945 (76.3) | 305 (73.8) | 0.672 | 0.440 | 0.596 | 0.861 |
|  |  | GA | 276 (22.3) | 102 (24.7) |  |  |  |  |
|  |  | AA | 18 (1.5) | 6 (1.5) |  |  |  |  |
| ***LDLR*** | rs6511721 | AA | 678 (54.7) | 229 (55.4) | 0.097 | 0.559 | 0.372 | 0.691 |
|  |  | GA | 492 (39.7) | 154 (37.3) |  |  |  |  |
|  |  | GG | 69 (5.6) | 30 (7.3) |  |  |  |  |
|  | rs2738446 | CC | 869 (70.1) | 280 (67.8) | 0.962 | 0.469 | 0.574 | 0.861 |
|  |  | CG | 337 (27.2) | 119 (28.8) |  |  |  |  |
|  |  | GG | 33 (2.7) | 14 (3.4) |  |  |  |  |
|  | rs2738459 | AA | 738 (59.6) | 220 (53.3) | < 0.001 | 0.296 | – | – |
|  |  | AC | 392 (31.6) | 157 (38.0) |  |  |  |  |
|  |  | CC | 109 (8.8) | 36 (8.7) |  |  |  |  |

H–W *P* < 0.05 means the SNP failed the HWE test. SNP = single-nucleotide polymorphism, PMI = premature myocardial infarction, PCSK9 = proprotein convertase subtilisin/kexin type 9, APOB = apolipoprotein B, LDLR = low-density lipoprotein receptor.

**Table S2. Gene–environment interaction between the rs151193009 variant of *PCSK9* and traditional risk factors for CHD**

| **Risk factors** | **Genotype** | **Control group, n (%)** | **PMI group, n (%)** | ***OR* (95% *CI*)** | ***P* value** | ***SI* (95% *CI*)** |
| --- | --- | --- | --- | --- | --- | --- |
| **Hypertension** |  |  |  |  |  |  |
| No | CC | 774 (62.5) | 172 (41.6) | 1.000 |  | 0.413 (0.005–30.095) |
| No | CT + TT | 30 (2.4) | 1 (0.2) | 0.150 (0.020–1.107) | 0.063 |  |
| Yes | CC | 414 (33.4) | 234 (56.7) | 2.543 (2.202–3.202) | < 0.001 |  |
| Yes | CT + TT | 21 (1.7) | 6 (1.5) | 1.286 (0.511–3.233) | 0.593 |  |
| **Diabetes** |  |  |  |  |  |  |
| No | CC | 1075 (86.8) | 302 (73.1) | 1.000 |  | 2.769 (0.149–51.471) |
| No | CT + TT | 49 (4.0) | 4 (1.0) | 0.291 (0.104–0.812) | 0.018 |  |
| Yes | CC | 113 (9.1) | 104 (25.2) | 3.276 (2.438–4.402) | < 0.001 |  |
| Yes | CT + TT | 2 (0.2) | 3 (0.7) | 5.339 (0.888–32.100) | 0.067 |  |
| **Smoking** |  |  |  |  |  |  |
| No | CC | 829 (66.9) | 130 (31.5) | 1.000 |  | 0.120 (0.001–224.020) |
| No | CT + TT | 33 (2.7) | 3 (0.7) | 0.580 (0.175–1.918) | 0.372 |  |
| Yes | CC | 359 (29.0) | 276 (66.8) | 4.903 (3.847–6.248) | < 0.001 |  |
| Yes | CT + TT | 18 (1.5) | 4 (1.0) | 1.417 (0.472–4.253) | 0.534 |  |

*SI* > 1 indicates that there is a synergistic interaction between the variant and the risk factor for CHD, *SI* = 1 indicates that there is no interaction between the variant and the risk factor for CHD, and *SI* < 1 indicates that there is an antagonistic interaction between the variant and the risk factor for CHD. PMI = premature myocardial infarction, OR = odds ratio, CI = confidence interval, SI = synergy index.

**Table S3. The relationship between the rs151193009 variant and the levels of TC and LDL-C in this study**

|  | **TC (mmol/L)** | | | **LDL-C (mmol/L)** | | |
| --- | --- | --- | --- | --- | --- | --- |
|  | **Variant carriers** | **Non-variant carriers** | ***P* value** | **Variant carriers** | **Non-variant carriers** | ***P* value** |
| **Cohort 1** | 3.85± 1.13 | 4.17 ± 1.22 | 0.041 | 2.33 ± 1.22 | 2.75 ± 0.91 | 0.009 |
| **Cohort 2** | 4.26 ± 0.77 | 4.56 ± 1.05 | 0.011 | 2.51 ± 0.70 | 2.88 ± 1.12 | 0.001 |

TC = total cholesterol, LDL-C = low-density lipoprotein cholesterol.
